# The Role of Probiotics in Nonalcoholic Fatty Liver Disease: A New Insight into Therapeutic Strategies

**DOI:** 10.3390/nu11112642

**Published:** 2019-11-04

**Authors:** Marica Meroni, Miriam Longo, Paola Dongiovanni

**Affiliations:** 1 General Medicine and Metabolic Diseases, Fondazione IRCCS Ca’ Granda Ospedale Maggiore Policlinico, Pad. Granelli, via F Sforza 35, 20122 Milan, Italy; maricameroni11@gmail.com (M.M.); longo.miriam92@gmail.com (M.L.); 2 Department of Pathophysiology and Transplantation, Università degli Studi di Milano, 20122 Milano, Italy; 3 Department of Clinical Sciences and Community Health, Università degli Studi di Milano, 20122 Milano, Italy

**Keywords:** nonalcoholic fatty liver disease, gut microbiota, gut–liver axis, leaky gut, intestinal permeability, tight junctions, endotoxemia, probiotics

## Abstract

Nonalcoholic fatty liver disease (NAFLD) encompasses a broad spectrum of pathological hepatic conditions ranging from simple steatosis to nonalcoholic steatohepatitis (NASH), which may predispose to liver cirrhosis and hepatocellular carcinoma (HCC). Due to the epidemic obesity, NAFLD is representing a global health issue and the leading cause of liver damage worldwide. The pathogenesis of NAFLD is closely related to insulin resistance (IR), adiposity and physical inactivity as well as genetic and epigenetic factors corroborate to the development and progression of hepatic steatosis and liver injury. Emerging evidence has outlined the implication of gut microbiota and gut-derived endotoxins as actively contributors to NAFLD pathophysiology probably due to the tight anatomo-functional crosstalk between the gut and the liver. Obesity, nutrition and environmental factors might alter intestinal permeability producing a favorable micro-environment for bacterial overgrowth, mucosal inflammation and translocation of both invasive pathogens and harmful byproducts, which, in turn, influence hepatic fat composition and exacerbated pro-inflammatory and fibrotic processes. To date, no therapeutic interventions are available for NAFLD prevention and management, except for modifications in lifestyle, diet and physical exercise even though they show discouraging results due to the poor compliance of patients. The premise of this review is to discuss the role of gut–liver axis in NAFLD and emphasize the beneficial effects of probiotics on gut microbiota composition as a novel attractive therapeutic strategy to introduce in clinical practice.

## 1. Introduction

The global burden of nonalcoholic fatty liver disease (NAFLD) as the leading cause of chronic liver disorders represents a major concern for public health. It encompasses a wide spectrum of hepatic conditions ranging from simple steatosis, a benign manifestation characterized by lipid accumulation exceeding 5% of liver weight excluding other etiological causes, to a more severe form, such as nonalcoholic steatohepatitis (NASH), fibrosis, cirrhosis and hepatocellular carcinoma (HCC) [1,2]. NAFLD is broadly spread in Western countries, affecting between 20% and 40% of the adult population, possibly due to the epidemics of obesity and type 2 diabetes mellitus (T2DM) [3].

The pathogenesis of NAFLD is closely intertwined with increased adiposity, insulin resistance (IR) and dyslipidemia [4]. Dietary factors such as excessive caloric intake, fructose and physical inactivity represent other risk factors for this condition [5]. Furthermore, the inter-individual variability in NAFLD phenotype may be at least in part attributed to genetics. Single nucleotide polymorphisms (SNPs) in proteins regulating hepatocellular lipid handling, including Patatin-like Phospholipase Domain-containing 3 (PNPLA3), Transmembrane 6 Superfamily Member 2 (TM6SF2) and Membrane Bound O-acyltransferase Domain-containing 7 (MBOAT7), have been associated to NAFLD predisposition and progression towards NASH and fibrosis [6]. However, less than 10% of inherited variability is explained by these common variants. Many of the phenotypic differences may also result from gene-environment interactions, referred to as epigenetics, a hereditable but reversible phenomenon that affects gene expression without modifying DNA sequence, such as alterations of DNA nucleotides (i.e., methylation), modifications of histones and regulation of transcription by altering mRNA stability through small RNA molecules such as microRNAs (miRNAs) [7,8].

Therefore, as a complex disease, the pathophysiology of NAFLD is not completely elucidated and may be simultaneously influenced by multiple parallel hits including IR, oxidative stress, inflammation, epigenetic modifiers and many others. Among the plethora of risk factors, recent evidence has pointed out to the role of gut microbiota and its metabolites in the pathophysiology of alcoholic fatty liver disease (ALD) and NAFLD [9,10]. Indeed, qualitative and quantitative changes in gut microbiome composition (referred to as ‘dysbiosis’) and derangement in the gut–liver axis that favors viable gut-derived bacteria and endotoxins translocation into the bloodstream have emerged to be independently associated to the development of NAFLD and its progression to NASH and HCC. Thus, species-specific microbial communities might profile NAFLD stages [11,12,13,14,15], possibly enabling the intestinal flora modulation a diagnostic strategy and an eventual therapeutic intervention in the personalized NAFLD management. Currently, liver biopsy remains the gold standard procedure for diagnosis of NASH and no medications have been approved for the treatment of NAFLD patients except for modifications in lifestyle, nutrition and physical exercise and weight loss [16,17].

For this reason, this review aimed to focus on the relevance of gut microbiota dysregulation in the development and progression of NAFLD and its pivotal role as non-invasive biomarkers and therapeutic target in the tailored NAFLD clinical management. Therefore, we will highlight the use of probiotics, emphasizing their beneficial effects on dysbiosis as a potential therapeutic approach to introduce in clinical practice.

## 2. Insight into the Gut Microbiota in NAFLD

The human gastrointestinal lumen is the physiological habitat for more than 100 trillion microorganisms, which is approximately ten-times the number of somatic cells in the human body, hosting a wide variety of microbial species (archaea, fungi, yeast, bacteria and viruses) [16]. The gut microflora is a large reservoir of commensal microbes that live synergistically with the host and provide biological and metabolic functions benefiting the host. It includes more than 160 different bacterial species, including anaerobes and they carry more than three million unique genes [17,18]. Among them, bacteria predominate with the phyla of the Gram-positive Firmicutes and Gram-negative Bacteroidetes, mainly involved in the short-chain fatty acids (SCFAs), i.e., acetate, butyrate and propionate and hydrogen production, respectively. The other phyla are represented by Actinobacteria, Fusobacteria, Proteobacteria and Verrucomicrobia [19,20,21]. The precise function of the intestinal flora remains largely uncharted. However, it processes complexed and indigestible polysaccharides to SCFAs, providing energy to the host and it also participates in vitamin (i.e., vitamin B and K), bile acid and amino acid synthesis, drug and toxin metabolism and intestinal barrier preservation. In particular, the term ‘dysbiosis’ indicates all imbalances between beneficial and pathogen bacteria or modifications in intestinal flora taxonomic composition and/or function [22]. Perturbations in intestinal microbiota homeostasis has been already described not only in NAFLD, but also in ALD [10], T2DM [23], obesity [24,25] and many other diseases [26,27,28,29].

Along the gastrointestinal tract (GIT) from the mouth to colon, the bacterial concentration and composition is strikingly diverse (increasing from stomach to colon), showing even higher variability depending on the age, lifestyle, medications and diets. Indeed, a diet enriched in animal fat and sugars as well as the Western diet may predispose to bacterial overgrowth, immune system activation and mucosal inflammation both in preclinical [30,31] and clinical studies [32,33].

Several approaches have been developed to study the intestinal flora community diversity, exploiting quantitative real time polymerase chain reaction (qRT-PCR), sequencing of the 16S ribosomal RNA (rRNA) gene through next-generation DNA sequencing or partial 16S rRNA sequencing in the V6–V8 region through pyrosequencing, excepting for Enterobacteriaceae and Enterococcaceae families [34]. These tools provide information about the abundance and the taxonomy of microbial species in mucosa-associated colonic tissue biopsies and in fecal samples. All these techniques are also coupled with the more expensive metagenomics or metatranscriptomics shotgun approaches [35]. Nonetheless, to study the host-microbiome interactions, intestinal, systemic, uric, and fecal bacterial-products and metabolites, such as bile acids, SCFAs and endotoxins, can be assessed by using proteomic and metabolomic methods and may represent diagnostic noninvasive markers, reflecting the microbiota composition [36].

### 2.1. Preclinical Models of Microbiota Alterations in NAFLD

Several lines of evidence indicate that intestinal flora composition and function play a paramount role in the development of obesity and NAFLD [37] and preclinical models are particularly worthwhile in the understanding of the implications of enteric dysbiosis and bacterial overgrowth in the pathophysiology of these diseases. Indeed, the unbalanced intestinal flora may exert a detrimental effect on the host nutrient metabolism thus facilitating overweight and fatty liver onset. In keeping with this finding, germ-free mice are protected against diet-induced obesity and display less severe fat accumulation into the liver upon Western diet, supporting the crucial role of intestinal flora in NAFLD pathogenesis [37,38]. In these mice, the protection against obesity and hepatic steatosis may be possibly explained by higher circulating levels of Angiopoietin-like 4 (ANGPTL4), a serum hormone that impaired fat storage in adipocytes, muscles and heart and by the ability of microbiota to cleave and ferments complex dietary plant polysaccharides [39]. In physiological conditions, indeed, SCFAs and monosaccharides, resulting from polysaccharides digestion, are absorbed by the host and delivered to the liver where they are converted to complex lipids. Therefore, the over-representation of specific classes of bacteria facilitates the catabolism of absorbed nutrients and through the regulation of the expression of host genes, it promotes energy harvest and storage in adipocytes favoring the progressive development of obesity and hepatic steatosis [25]. According to this notion, microbiota transplantation from mice with diet-induced obesity to lean germ-free recipients promoted greater fat deposition compared to mice transplanted intestinal flora isolated from lean donors, suggesting that obesity-associated gut microbiota is responsible for the transmission of the ability to promote fat storage [25]. Furthermore, maternal obesity exacerbated the risk of hepatic disorders onset in the offspring [40]. Indeed, germ-free mice colonized with stool microbes isolated from 2-week-old infants born to obese mothers displayed endothelial reticulum (ER) stress, activation of innate immunity and periportal inflammation, recapitulating the histological pattern of the childhood NAFLD [40]. The exposure of these mice to a Western diet promoted an excessive weight gain, further precipitating NAFLD onset [40]. Finally, germ-free mice were also reported to be resistant to high-fat diet (HFD)-induced IR, showing enhanced hepatic and adipose tissue insulin sensitivity and fecal lipid excretion [41], revealing that insulin resistance index (HOMA-IR) is transmissible [38].

Multiple preclinical models are currently exploited to study NAFLD development and progression to advanced stages of liver diseases, resembling human hepatic lesions [42] and recently, these models have been pointed out to deeply investigate the alterations of gut microbiota composition in the context of liver injury.

An unbalance of bacterial species was reported in Leptin deficient mice (*Lep*^ob/ob^), a genetic model of obesity, IR and NAFLD, which had enhanced Firmicutes and lower Bacteroides levels, alterations that have been associated with obesity and subsequent chronic liver diseases [24,25]. Indeed, DNA sequencing of cecal microbiota of *Lep^ob/ob^* indicated that the obesity-associated gut microbiome had an increased capacity for fermenting polysaccharides respect to the lean-associated one, due to the enhanced prevalence of Firmicutes [24].

As well as *Lep^ob/ob^* mice, even HFD-fed mice carried a peculiar gut microbiota signature, which markedly impacted on obesity, IR and lipid metabolism in the liver [38]. Indeed, Le Roy and coworker demonstrated that gut microbiota play a causative role in the susceptibility to develop NAFLD features including hyperglycemia, IR and steatosis, in response to the HFD challenge and that the propensity to develop NAFLD is transmissible by means gut microbiota transplantation [38]. The authors revealed that alterations in taxonomic composition, such as decreased quantity of Bacteroidetes and increased levels of Firmicutes, were responsible of NAFLD development, similarly to what occurs in obesity [25]. Specifically, *Barnesiella intestinihominis,* which was previously related to increased hepatic steatosis and inflammation [43], was found increased in HFD mice, whereas *Bacteroides vulgatus* was reduced, as well as in patients affected by diabetes [44]. Nonetheless, the suppression of intestinal flora via chronic oral administration of antibiotics attenuated hepatic inflammation and fibrosis in HFD mice, as a result of the decrease in portal secondary bile acids, supporting the notion that a causal link between gut microbiota and liver damage exists [45].

Derangement in gut microbiota composition was even described in mice fed methionine-choline deficient diet (MCD), a dietary model to study NAFLD/NASH in absence of obesity and IR, specifically, harboring a marked decrease in the abundance of *Alistipes* and the (*Eubacterium*) *coprostanoligenes* group and a parallel increase in Ruminococcaceae [46].

Gut dysbiosis has been also causally linked to the pathogenesis of cirrhosis and progression to end-stage liver disease [47]. Depletion of host microflora after gut sterilization could suppress tumor formation, reducing impressively size and number of nodules in diethylnitrosamine (DEN)-induced HCC [48]. In line with these findings, Dapito and colleagues reported that mice grown in germ-free conditions developed smaller and fewer HCC, and treatment with low dose of endotoxins reverted this situation [49]. These studies demonstrated that gut microbiota and Toll-like receptor 4 (TLR4) are required for the tumorigenesis promotion, mediating proliferation and prevention of apoptosis [48,49].

### 2.2. Human Gut Microbiota in NAFLD

An increasing number of studies have demonstrated that bacterial overgrowth may adversely impact metabolic processes and immune responses, favoring obesity and obesity-related comorbidities, including NAFLD and IR [50]. However, the precise characterization of dysbiosis in the whole spectrum of NAFLD lesions has still been unexplored. In 35 consecutive patients with biopsy-proven NAFLD, Miele et al. demonstrated that NAFLD patients had a significantly increased gut permeability compared to healthy subjects and the prevalence of small intestinal bacterial overgrowth strictly correlated with the severity of steatosis, but not with lobular inflammation, ballooning and fibrosis [51]. Even more, patients affected by NASH displayed intestinal bacterial overgrowth, as assessed by the (14)C-D-xylose-lactulose breath test, increased endotoxins and inflammatory cytokines into the blood circulation [52]. Thus, the degree of NAFLD is correlated to dysbiosis and to modifications of metabolic properties of intestinal flora [53].

The main bacterial composition modifications observed in NAFLD patients are represented by an enrichment in Proteobacteria, Enterobacteriaceae, Lachnospiraceae, *Escherichia* and Bacteroidetes. However, there are several discrepancies in the proportion of the latters between the studies and the results are heterogeneous, mainly due to the presence of obesity and metabolic syndrome as confounders [54,55,56].

An unbalance in the ratio between Bacteroidetes and Firmicutes has been reported by Zhu and colleagues in fecal samples of obese and NASH children [57]. In particular, they assessed the composition of gut bacterial communities of 22 biopsy-proven NASH children, of 25 obese subjects and 16 healthy controls by 16S ribosomal RNA pyrosequencing and they revealed an enhanced abundance of Bacteroidetes and a decreased number of Firmicutes in fecal samples of obese and NASH children. Even the levels of Actinobacteria were reduced in NASH individuals, conversely the quantity of Proteobacteria rose progressively from healthy to obese to NASH patients [57]. The main finding of this research is the presence of elevated blood levels of alcohol and the highest activities of alcohol dehydrogenases (ADHs) only in NASH children, due to the increased concentration of ethanol-producing bacteria from carbohydrate catabolism such as *Escherichia coli.* In physiological conditions, indeed, endogenous alcohol is constantly produced by the intestinal microbiota and rapidly removed from portal blood by hepatic ADHs, catalases and microsomal ethanol-oxidizing system [58,59]. In NASH-induced dysbiosis, conversely, the over-representation of alcohol-producing bacteria determined an exaggerated release of ethanol into the blood flow, further corroborating liver inflammation, reactive oxygen species (ROS) production via the Cytochrome P450 2E1 (CYP2E1) [57,60] and intestinal hyperpermeability [57].

Fecal dysbiosis and decreased quantity of Firmicutes has been also observed by Wong et al. [55] in 16 NASH patients compared to 22 controls. The authors showed the presence of lower fecal abundance of Faecalibacterium and Anaerosporobacter in these subjects but higher abundance of Parabacteroides and Allisonella [55]. Moreover, Sobhonslidsuk and coworkers highlighted an increase in the Bacteroidetes/Firmicutes ratio in 16 adult patients affected by NASH independently of age, body mass index (BMI), diabetes and medications [61]. In particular among the Bacteroidetes phylum, the *Bacteroides* and *Prevotella* genera are the most abundant in NASH subjects [61]. Conversely, Mouzaki and collaborators revealed a reduction in Bacteroidetes and higher levels of fecal *Clostridium coccoides* in 22 NASH subjects compared to 17 healthy subjects and 11 simple steatosis, thus facilitating the growth of other bacterial species and the override energy intake from dietary fat [24,25,62]. The variety in the proportion of Bacteroidetes and Firmicutes observed by Zhu and colleagues [57] and Mouzaki et al. [54] may reflect the diversity in age, BMI, environmental and dietary factors of the two study cohorts. Therefore, to rule out the impact of obesity on gut microbiota composition, Wang B. and colleagues sought to identify the variability in fecal microbiota composition between non-obese adult individuals with and without NAFLD (43 NAFLD vs. 83 healthy controls) [63]. They demonstrated that adult non-obese NAFLD patients harbored 20% more phylum Bacteroidetes and 24% less Firmicutes, showing a significant correlation of metabolic markers with the disturbed microbiota in NAFLD [63]. Hence, the prevalence of Firmicutes is considered a fingerprint of obesity-associated NAFLD, whereas the Bacteroides override is related to ‘lean’ NAFLD.

Overwhelming evidence provided by Loomba et al. suggested the presence of gut microbiota-derived signature, which predicts the presence of advanced fibrosis in NAFLD patients [64]. Through a whole-genome shotgun sequencing approach on stool samples, the authors analyzed the bacteria taxonomic composition of 86 biopsy-proven NAFLD of whom 72 had mild fibrosis and 14 had advanced fibrosis (stages 3/4). They identified 37 different bacterial species, which enabled us to distinguish mild and advanced fibrosis in NAFLD patients and they showed that advanced fibrosis is characterized by an exasperation of Proteobacteria and *Escherichia coli* along with a decrease in Firmicutes [64]. The same research group, in a very recent paper, identified the specific intestinal microflora profile of NAFLD cirrhotic patients, determining a panel of 27 fecal bacteria that may discriminate NAFLD cirrhosis using a random forest classifier model [65]. Besides, Boursier et al. determined the presence of rising quantity of Ruminococcus in NASH patients affected by advanced fibrosis, while *Prevotella* abundance was decreased [53] and higher counts of *Escherichia coli* and *Staphylococcus* have been found in stool samples of subjects with mild encephalopathy and cirrhosis [66]. Quantitative metagenomic analyses identified 75,245 genes that differ cirrhotic patients from healthy individuals [67], showing fewer Bacteroidetes but higher levels of Proteobacteria and Fusobacteria [67]. Schierwagen and colleagues focused on the assessment of the circulating microbiome in the portal vein of seven patients with decompensated cirrhosis, during the implantation of a transjugular intrahepatic portosystemic shunt and they demonstrated that 65 genera belonging to four phyla, predominantly Proteobacteria, were strictly correlated with cytokines secretion [68].

Finally, addressing to fecal microbiota diversity in HCC patients, Ren Z. and coworkers revealed an enrichment in the phylum Actinobacteria and in 13 genera, including *Gemmiger* and *Parabacteroides* in fecal samples of 75 HCC patients compared to 40 cirrhotic ones. In particular, butyrate-producing genera were decreased, while lipopolysaccharides (LPS)-producing genera were increased in early stage of HCC [69].

Collectively, these findings suggest that manipulation of intestinal microflora may be a strategy to prevent or treat NAFLD and metabolic syndrome features.

## 3. Gut–Liver Axis: New Awareness in NAFLD Pathogenesis and Progression

The gut–liver axis has many implications in NAFLD onset as the major contributor of the intestinal dysbiosis, possibly due to the tight anatomo-functional crosstalk of the two organs. The liver is perpetually exposed to gut microbial end-products and nutrients via the portal vein (70% of blood supply) and, in turn, participates to bacterial composition through bile acids cycling released into the duodenum lumen with the enterohepatic circulation [10]. Alongside, the gut microbiome composition is crucial to modulate innate and adaptive immune response both locally and systemically, facilitating host defense against pathogens.

The bowel wall plays an essential role as a selective barrier that regulates the bidirectional flux between the gut and the liver, since it is constituted by tight and adherent junctions (occludins, claudins and Zonula Occludens 1 (ZO-1)) and desmosomes, which hold together the epithelial cells. Furthermore, it exerts many immunological functions as it is constituted by multiple layers and specialized cells, such as Goblet, Paneth and plasma cells secreting mucus, antimicrobial peptides (i.e., defensins, lysozyme and c-lectin Reg3b/g) and Immunoglobulin A (IgA), respectively. Together they protect the host from invasive pathogens and avoid bacterial overgrowth and systemic translocation [10]. The excessive erosion of the protective mucus layer as well as the reduction of antimicrobial mediators has been associated with translocation of pathogenic microorganisms in both preclinical and human studies [70,71].

Disturbance of the intestinal barrier integrity, a phenomenon known as leaky gut, along with shifting in metabolic function of gut microbiota, are frequently present in patients with NAFLD-related dysbiosis [51,72] and correlate with NAFLD severity. Indeed, a relative abundance in Bacteroides and Ruminococcus have been independently associated with NASH and fibrosis [73]. As a consequence of enhanced gut permeability, much more bacteria and potentially harmful byproducts translocate into circulation and reach the liver thus contributing to the increase of circulating gut-derived toxins (endotoxemia) and the establishment of chronic low-grade inflammatory state that features metabolic disorders such as obesity and NAFLD [74,75]. Several endogenous molecules as ethanol, ammonia and acetaldehyde, whose circulating increased levels result from dysbiotic microbiota (i.e., *Escherichia coli* abundance), are able to stimulate hepatic Kupffer cells to produce pro-inflammatory cytokines with similar mechanisms occurring in ALD [10,57]. Likewise, LPS and peptidoglycans derived from Gram-negative and Gram-positive bacteria walls are the most representative pathogen-associated molecular patterns (PAMPs), which activate Toll-like receptors (TLRs) signaling. In particular, LPS-induced TLR-4 cascade in hepatocytes, Kupffer cells and hepatic stellate cells (HSCs) leading to elevated systemic levels of tumor necrosis factor alpha (TNF-α) and interleukin 6 (IL6) via nuclear receptor kappa B (NF-kB) thus promoting IR, inflammation and fibrosis [9,76]. Otherwise, circulating free fatty acids (FFAs), whose levels are commonly higher in NAFLD, may independently stimulate TLR4 and TLR2 inflammatory pathways [77,78]. Furthermore, peptidoglycans and damage-associated molecular patterns (DAMPs) contribute to liver damage through the crosstalk between TLRs (e.g., TLR2 and TLR5) and inflammasome via intracellular nucleotide-binding and oligomerization domain (NOD)-like receptors (NLRs), which increase IL1 and IL8 production in hepatocytes, Kupffer cells and HSCs [60].

Moreover, alteration of gut microflora communities contributes to liver pathology and disruption of intestinal barrier integrity. For instance, dysbiosis may affect lipid metabolism and trafficking in both liver and adipose tissue by upregulating lipogenic enzymes or lipoprotein lipase (LPL) thus participating to obesity and steatosis development. Interestingly, several intestinal bacteria species dampen the production of the Fating-Induced Adipocyte Factor (FIAF), whose downregulation is associated with increased adiposity and hepatic de novo lipogenesis [79]. Enrichment in Cytophaga–Flavobacter–Bacteroides phyla influences the development of fatty liver and hepatic inflammation favoring IL7 release from T-helper cells (Th17) [80]. Dietary choline is further metabolized by enteric bacteria in trimethylamine and then it is converted in the hepatotoxic trimethylamine N-oxide (TMAO) end-product. Indeed, choline shortage or increased TMAO production have been associated with higher levels of Gram-negative Gammaproteobacteria and Erysipelotrichi and with steatosis since its levels are crucial to favor very-low density lipoprotein (VLDL) assembly and secretion.

Microbial SCFAs may affect the intestinal barrier integrity and mucosal immune tolerance raising levels of intestinal SCFAs-producing species strengthen barrier integrity supporting tight junctions and mucins production and operating as energy source for intestinal mucosal cells [81,82]. For example, the reduction of produce butyric acid, produced by *Faecalibacterium prausnitzii*, weakens the few connections between intestinal epithelial cells, by decreasing the expression of the tight junction proteins and mucins. The restoration of the physiological abundance of microorganisms-producing butyrate, in turn, may ameliorate the gut high permeability and systemic inflammation [83].

**Figure 1:**
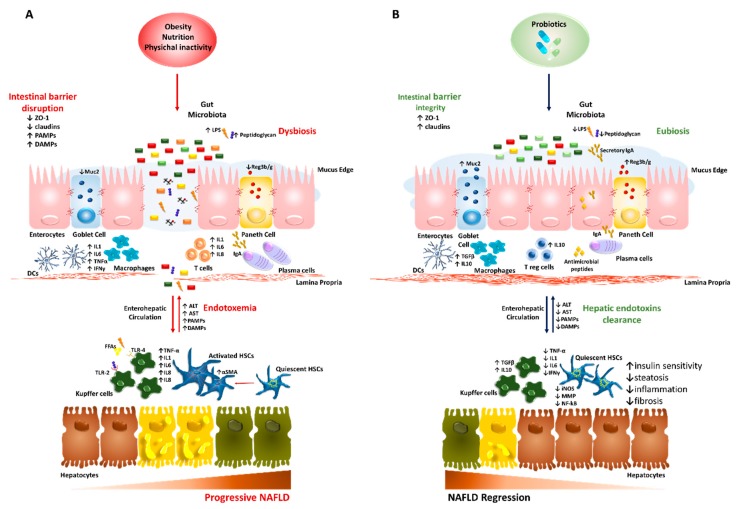
Role of the gut–liver axis in progressive nonalcoholic fatty liver disease (NAFLD) and probiotic-related beneficial mechanisms. We summarized the main abnormalities involved in dysbiotic NAFLD and the impact of probiotic treatment on the gut–liver axis. (**A**) Obesity, diet and physical inactivity favor hepatic steatosis, disruption of the intestinal barrier and alter microflora taxonomic composition (dysbiosis). This condition may promote gut hypermeability through loosening tight junction proteins (ZO-1 and claudins), thinning mucus layer (Muc2), reducing antimicrobial peptides and IgA secretion. The leaky gut stimulates a pro-inflammatory local milieu, recruiting macrophages, dendritic cells (DCs), T CD4+ and T CD8+ as well as promoting phenotype switch of B cells into plasma cells. This pathologic microenvironment facilitates the systemic translocation of pathogenic bacteria and gut-derived pathogen-associated molecular patterns (PAMPs)/damage-associated molecular patterns (DAMPs; such as lipopolysaccharides (LPS) and peptidoglycans). Upon arrival to the liver via portal vein, PAMPs/DAMPs and high levels of free fatty acids (FFAs) activate inflammatory response in hepatocytes, Kupffer cells and hepatic stellate cells (HSCs) through Toll-like receptors (TLRs) cascade, which enhances the release of cytokines and chemokines (such as TNFα, IL1, IL6, IL8 and IFN-γ) and worsens liver damage. (**B**) Probiotics may restore intestinal barrier integrity, positively acting on ZO-1 expression, mucus thickness and commensal bacteria proportion (eubiosis). Moreover, they participate to the shutdown of bowel inflammation, enrolling T regulatory cells, DCs and macrophages to secrete anti-inflammatory cytokines (TGF-β and IL10). In the liver, the reduction of endotoxemia halts hepatic damage, as shown by lower aminotransferases (ALT and AST) and contributes to the recovery of the hepatic functions, affecting the lipid composition of fatty-laden hepatocytes, favoring endotoxins clearance, as well as negatively impacting on inflammatory and fibrogenic processes (i.e., lower iNOS, MMP and NF-kB).

The molecular features of the gut–liver axis in NAFLD/NASH are schematically represented in Figure 1A.

### 3.1. Bile Acids Pool: A Fine-Tuning Regulator of Intestinal Barrier Integrity

Emerging evidence has suggested that alterations in bile acids metabolism are associated with chronic liver diseases and comorbidities, i.e., development of cholestasis. The liver synthetizes primary bile acids, which first accumulate in the gallbladder and then are released in the duodenum lumen where they are converted in secondary bile acids by the gut microbiota and favor lipid solubilization, emulsification and absorption. In addition, bile acids act as signaling molecules (i.e., deoxycholic acid (DCA) and di-hydroxy chenodeoxycholic acid (CDCA)) as they activate the intestinal Farnesoid X Receptor (FXR), leading to the release of Fibroblast Growth Factor 19 (FGF19), which can modulate gut barrier integrity, and β-Klotho in the bloodstream [10]. Both FGF19 and β-Klotho can downregulate bile acids synthesis in the liver by inhibiting cholesterol 7-α hydroxylase 1 (Cyp7A1) [84]. The enhanced production of bile acids pool can also stimulate Takeda G-protein-coupled receptor 5 (TGR5) to activate the proinflammatory cascade on Kupffer cells surface [85]. Indeed, patients with NASH showed elevated levels of cytotoxic bile acids in the liver, serum, stool and urine, which may worsen liver damage to cirrhosis [86,87,88]. To date, many FXR agonists with hepatoprotective properties (i.e., obeticholic acids) have been proposed as they are able to reduce hepatic steatosis and necroinflammation [88,89]. However, the prolonged exposure to bile acids analogues is associated with severe side effects. Therefore, alternative approaches, such as ω-3 long-chain polyunsaturated fatty acids administration and/or combined to gut microbiota modulation, which may have the advantage to adjust bile acid pool, is still under investigations for the treatment of NAFLD [90,91].

### 3.2. Features of the Gut–Liver Axis in NAFLD and NASH

The implication of the gut–liver axis in the susceptibility of NAFLD has been widely investigated in both preclinical and human studies, although it remains a matter of debate. Nevertheless, new insights in the pathogenesis and progression of NAFLD have been attributed to the permeable gut barrier as it constitutes a favorable microenvironment for bacteria overgrowth, promotes endotoxemia and contributes to chronic liver damage in response to endogenous or exogenous cofactors, such as dietary pattern and lifestyle. Indeed, fatty liver is highly prevalent in obese patients and the 20%–30% of pathologically obese individuals show histological signs of necroinflammation and fibrosis [92,93], possibly even for diet-induced derangement of barrier integrity.

It has been demonstrated that the interaction between HFD and enteric bacteria promote intestinal inflammation through TNF-α production and NF-kB activation, a mechanism that precedes IR development in mice [94]. In NAFLD rats feeding high sucrose and high fat (HSHF) diet, Zhou et al. showed that animals exhibited damaged villous of the intestinal epithelium and low-grade inflammatory status due to increasing gut-derived endotoxins and inflammatory cytokines that translocate into the circulation [95]. Interestingly, Brun et al. revealed that leptin-deficient (*Lep^ob/ob^*) and hyperleptinemic (*Lep^db^*^/*db*^) obese mice displayed a dysmorphic mucosal barrier as demonstrated by a dramatic ZO-1 and tight junctions redistribution, and a remarkable release of IL1, IL6, TNF-α and Interferon (IFN-γ) in the portal vein [96]. Notably, sodium butyrate supplementation to HFD fed mice improved gut mucosa restoring intestinal ZO-1 levels and favoring abundances of the beneficial bacteria Christensenellaceae, Blautia and Lactobacillus [97]. Butyrate further impacts on liver damage, strongly reducing hepatic fat accumulation as well as markers of inflammation and fibrosis [97]. Nonetheless, HSCs isolated from *Lep^ob/ob^* and *Lep^db^*^/*db*^ livers chronically exposed to LPS showed a pro-inflammatory and pro-fibrotic phenotype compared to HSCs isolated from lean mice, supporting that increased intestinal permeability may participate to the development and progression of obesity-related NASH [96].

Chronic fructose intake has been associated with loss of tight junction proteins and lower SCFA-producing agents, which foster PAMPs translocation, increase numbers of macrophages in the liver and activate TLR1-9 and myeloid differentiation factor 88 (Myd88)-dependent proinflammatory pathways in mice [98]. Similarly, acute and chronic high fructose consumption exacerbated endotoxemia in pediatric NAFLD and even correlates with markers of IR and liver inflammation [99]. Noteworthy, the G protein-coupled chemokine receptor CX3CR1 protects mice from steatohepatitis induced with HFD or MCD diet as it maintains intestinal homeostasis and barrier integrity [100].

Finally, in a model of early NASH induced with high glucose/fructose diet (HFGFD), the authors observed that rats developed portal hypertension, a severe complication of liver cirrhosis. Hence, they investigated whether enteric dysbiosis could modulate endothelial and hepatic functions. Notably, HFGFD-fed mice were enriched in Firmicutes rather than Bacteroides strains and selectively activated intestinal FXR thus suggesting that changes in intestinal microbiota communities impair bile acid metabolism, which, in turn, may be a driver of NASH-related complications [101].

### 3.3. Features of the Gut–Liver Axis in Cirrhosis and HCC

The aforementioned mechanisms further influence liver damage progression, possibly leading to advanced fibrosis and cirrhosis, the end-stage of chronic liver disease and the leading cause of liver failure and HCC. Activated HSCs show high LPS-induced TLR4 responsiveness thus repeatedly enhancing pro-fibrotic processes that compromise immune defense and toxins clearance from hepatic tissue supporting cirrhosis development [102]. Moreover, LPS/TLR4 signaling plays a critical role to induce hepatocarcinogenesis by promoting a senescence-associated secretory phenotype (SASP) in HSCs and stimulating chemoattractant cytokines production from HSCs and monocytes. Bacterial translocation even participates to hemodynamic complications linked to cirrhosis, such as hepatic encephalopathy, variceal bleeding and portal hypertension. Recently, Sorribas et al. showed that cirrhotic mice reduced mucus thickness and lost Goblet cells as well as mucins expression, allowing bacterial overgrowth and the pathological translocation of *Escherichia coli* [103]. Assimakopoulos et al. firstly demonstrated that leaky gut exerts a pivotal role in human cirrhosis, highlighting the importance of occludin and claudin 1 downregulation in cirrhotic patients and even more in those with decompensated cirrhosis [104]. In addition, higher levels of IL6, nitric oxide (NO) and decreased transepithelial resistance have been associated with the presence of activated intestinal macrophages in cirrhotic patients [105].

Obesity-induced changes in gut microbiota composition may raise the amount of DCA that has been associated with SASP phenotype in HSCs, which enhance pro-inflammatory cytokines and tumor-promoting factors, as well as DNA damage and ROS production in the liver [106,107]. In an experimental model of HCC induced with DEN, alterations in gut permeability seems to be the primary hit leading to amplified tumorigenic response of the liver to LPS. Indeed, antibiotics regimen or TLR4 ablation mitigated tumor growth and multiplicity in mice [48]. Dapito et al. exposed C3H/HeJ and C3H/HeOuJ mice to a mixture of DEN and hepatotoxin carbon tetrachloride (CCL4), a model that resembles human microenvironment for HCC raise, and demonstrated that intestinal microbiota and TLRs promote liver cancer as a long-term consequence of chronic liver injury [49]. In a most recent study, Ponziani and collaborators investigated the gut microbiome profile and intestinal features of 21 NAFLD patients with both cirrhosis and HCC compared to 20 cirrhotic individuals without HCC and healthy controls. Although they found a similar degree of intestinal barrier dysfunction between HCC and cirrhotic subjects, systemic levels of IL8, IL13, Chemokine C-C motif Ligand 3 (CCL3), CCL4 and CCL5 significantly correlated with circulating activated monocytes in presence of HCC [108].

## 4. Probiotics: Cunning Double-Crossers Against Their Household

Current interventions for the management of NAFLD focused on dietary and lifestyle modifications, although the discouraging results due to the poor compliance of patients. In addition, hypolipidemic drugs, anti-TNFα, antioxidants and diabetes medications have been proposed for NAFLD/NASH, even though no pharmacological therapies or surgical procedures have been approved for the treatment of NAFLD. In the last decade, intensive efforts have been directed to develop new strategies targeting the gut–liver axis as it appears as an attractive converging point for the prevention of NAFLD onset and/or progression. Several approaches to modulate dysbiosis include 1) untargeted methods (diet, probiotics, prebiotics, antibiotics and fecal microbiota transplant (FMT)) or 2) microbiota-targeted therapy (MTT) which selectively target microbial and host metabolites [109].

The mechanisms by which unbalancing in gut microbiota participate to liver pathology remains still uncertain; however promising results on modulation of intestinal flora have been reported in several preclinical and human studies. Increasing efforts have been addressed to exploit the ability of probiotics to reverse gut dysbiosis and only recently they have been proposed as treatment of NAFLD.

Probiotics are defined as a “live microorganism that—when administered in adequate amounts—confer a health benefit on the host” by the World Health Organization/Food and Agriculture Organization (WHO/FAO). The criteria for the selection of probiotic strains are represented by the safety (i.e., absence of genes responsible for antibiotic resistance), functionality (i.e., resistance of lower pH in the stomach) and technological usability (i.e., high survival rate in finished products) [110]. Among them, commercialized *Streptococcus/Lactobacillus/Bifidobacteria* promote anti-inflammatory environment and help intestinal epithelium growth and survival as well as they may counteract the pathogenic bacteria by modulating immune system and host defense [111].

The present chapter would deeply highlight the most recent findings on health benefits gained with probiotic administration in the experimental models of NAFLD and in the clinical practice.

The probiotics benefits on the gut–liver axis in NAFLD/NASH are summarized in Figure 1B.

### 4.1. Probiotics in the Preclinical Studies of NAFLD

Numerous studies demonstrated that probiotics administration might attenuate NAFLD features in animal models [112,113,114,115,116]. The administration of VSL#3, a mixture of three genera of bacteria (a multistrain formulation composed by *Streptococcus, Thermophilus* and several species of *Bifidobacterium* and *Lactobacillus*) for 4 weeks to Lep*^ob/ob^* mice improved insulin sensitivity, total fatty acid content, serum alanine aminotransferase (ALT) levels and the histological spectrum of liver damage [112]. These improvements were mainly due to the reduction of Jun N-terminal kinase (JNK) and NF-kB activation and to the decreased expression of Uncoupling protein (UCP)-2 in Lep*^ob/ob^* mice exposed to VSL#3, sustaining the hypothesis that intestinal bacteria may regulate the activation of host signaling pathways interfering with hepatic insulin response and lipid metabolism [112].

Nonetheless, VSL#3 supplementation in HFD-challenged young rats, dampened the production of TNF-α, inducible nitric oxide synthase (iNOS), metalloproteinases (MMP) and Cyclooxygenase 2 (COX-2), as well as it improved lipid peroxidation markers, limiting oxidative and inflammatory damage in the liver [115]. These findings are even supported by Ma X. et al.’s study, in which VSL#3 probiotics exposure ameliorates IR, steatosis and pro-inflammatory cytokines secretion, hampering NF-kB activity in HFD-fed mice [113]. Furthermore, oral *Bifidobacterium longum* supplementation in HFD-fed rodents for 12 weeks reduced hepatic fat accumulation more than the administration of *Lactobacillus acidophilus*, irrespectively of intestinal permeability restoration [117]. In addition, *Lactobacillus johnsonii BS15* protected HFD mice from hepatic steatosis and hepatocyte apoptosis, exhibiting a positive effect on lipid peroxidation, sustaining the antioxidant defense system and improving mitochondria abnormalities [118]. The restoration of Bifidobacteria or *Akkermansia muciniphila* along with oligofructose implementation reduced endotoxemia, hepatic fat deposits and metabolic syndrome hallmarks in HFD mice [119]. Moreover, diabetic rats administered with *Akkermansia muciniphila* improve liver function, reduce gluco/lipotoxicity, alleviate oxidative stress, suppress inflammation and normalize intestine microbiota thereby ameliorating type 2 diabetes mellitus [120]. Similarly, nano-selenium-enriched *Bifidobacterium longum* delay the onset of streptozotocin-induced diabetes [121].

In a model of inherited dyslipidemia (ApoE-/-mice), the modulation of gut microbiota through VSL#3 mixture rescued hepatic and adipose tissue IR and counteracted atherosclerosis and NAFLD onset [122]. Specifically, VSL#3 reversed IR, prevented development of histologic features of mesenteric adipose tissue inflammation, NASH and reduced the extent of aortic plaques, through the modulation of Peroxisome Proliferator-Activated Receptor-γ (PPAR-γ), FXR and vitamin D receptor [122].

Velayudham and collaborators demonstrated that VSL#3 may even influence fibrosis development in MCD fed mice, favoring the reduction of collagens, MMPs and α-smooth muscle actin (α-SMA), not accompanied by the attenuation of hepatic steatosis and inflammation [116]. In agreement with Velayudham’s study, even Nardone and collaborators revealed the protective effect of *Lactobacillus paracasei* F19 (LP-F19), in an experimental model of ischemia-riperfusion in rats fed MCD [114]. Likewise, heat killed *Lactobacillus reuteri* GMLN-263 rescued hepatic and heart fibrosis, resetting the expression of pro-fibrotic markers, such as the transforming growth factor β (TGF-β) in HFD hamsters [123]. Alongside LP-F19 and GMLN-263, many other strains of *Lactobacillus,* i.e., *Lactobacillus reuteri* GMLN-13, have been demonstrated to attenuate the harmful impact of gut microbiota derangement in the contest of NAFLD and NAFLD-related comorbidities, such as hypertension, obesity, glucose intolerance, hyperglycemia, hyperinsulinemia, dyslipidemia, adipose tissue inflammation and oxidative stress [124,125,126,127].

In rodents, daily oral VSL#3 administration was next reported to be related to the restoration of enterocyte architecture and of intestinal barrier integrity by inducing mucus secretion, Muc2 colonic expression and ileal occludin levels, and avoiding viable microorganisms and bacterial products translocation into the bloodstream [128]. The expression of tight junction proteins (i.e., occludins, claudins and ZO-1) was forced by the supplementation with *Lactobacillus rhamnosus, Lactobacillus paracasei* and *Bifidobacterium adolescentis* [109]. Intriguingly, *Clostridium butyricum* strain MIYAIRI 588, a butyrate-producing probiotic, prevented the entire pathological spectrum of NAFLD, from steatosis to HCC in a rodent model of choline-deficient/L-amino acid-defined (CDAA)-diet, re-establishing the intestinal barrier integrity [129]. Besides, MIYAIRI 588 positively affects the development of IR, excessive triglyceride storage and attenuates serum endotoxin concentration, hepatic inflammation and oxidative stress [129]. Finally, *Lactobacillus johnsonii* La1 in combination with antioxidants prevented bacterial translocation and endotoxemia in a rat model of CCl4-induced cirrhosis, enlightening the idea to use probiotics and antioxidants as alternative strategy to antibiotics in the prevention of bacterial infections in cirrhotic patients [130].

Complexively, probiotics supplementation may positively intervene on the liver injuries induction in rodent models of NAFLD although most of the studies are addressed to prevent rather than treat diet-induced liver disease.

### 4.2. Use of Probiotics in Human NAFLD, Cirrhosis and HCC

Over the year, the experimental models of NAFLD have collected promising findings in the field of microbiome therapeutics thus providing greater awareness for the clinical evaluation of probiotics to overthrow NAFLD development and progression.

Vajro et al. conducted a double-blind study on 20 obese children with ultra-sonographic proved steatosis that were randomized for *Lactobacillus rhamnosus* GG or placebo for 8 weeks. Despite probiotic administration had no effect on adiposity and fatty liver, it has been suggested as treatment for hypertransaminasemia in obese children noncompliant to lifestyle interventions [131]. Similar findings of reduced ALT and aspartate aminotransferases (AST) have been observed in 30 adults affected by NASH exposed to *Lactobacillus acidophilus* compared to those receiving placebo [132].

The effects of probiotics as a combination of multistrains compound have reached better outcomes in randomized trials. Famouri et al. carried out a triple-blind trial on 64 obese children with sonographic NAFLD. Adolescents receiving a probiotic capsule of *Lactobacillus acidophilus* ATCC B3208, *Bifidobacterium lactis* DSMZ 32,269, *Bifidobacterium bifidum* ATCC SD6576 and *Lactobacillus rhamnosus* DSMZ 21,690 for 12 weeks showed significant reduction in ALT, lipid profile and intrahepatic fat content evaluated with ultrasound compared to placebo group. In a randomized placebo-controlled trials (RCT), Kobyliak et al. assessed the efficacy of “Symbiter”, containing 14 alive probiotic strains of *Lactobacillus + Lactococcus*, *Bifidobacterium*, *Propionibacterium* and *Acetobacter*, in NAFLD patients. The multiprobiotic cocktail ameliorated hepatic steatosis, aminotransferase activity, TNF-α and IL6 levels in patients with NAFLD [133]. Similarly, patients with histology-proven NASH randomly receiving Lepicol probiotic formula for 6 months attenuated intrahepatic triglycerides and reduced serum AST levels [134]. In a meta-analysis conducted by Ma et al., including 134 NAFLD/NASH from four randomized trials, the authors underlined that probiotic therapy with *Lactobacillus*, *Bifidobacterium* and *Streptococcus* beneficially impacts on hepatic fat content, total cholesterol, levels of aminotransferases and HOMA-IR [135]. Most recently, Gao and collaborators evaluated the efficacy of probiotic treatment in both pediatric and adult NAFLD including nine RCT with a total of 535 NAFLD cases [136]. They showed that probiotics improve the clinical outcomes of NAFLD patients, influencing insulin sensitivity and reducing TNF-α. However, probiotics ameliorated dyslipidemia only in Italian and Spanish population, suggesting that the effects of these molecules on high-density lipoprotein (HDL), low-density lipoprotein (LDL) and triglycerides might depend on ethnical background [136].

It has been demonstrated that VSL#3, the most studied multistrains probiotic, protects intestinal barrier integrity, dampens endotoxemia and oxidative/nitrosative stress thus favoring improvement in liver pathology in patients with different chronic liver diseases, among which 20 with ALD and 22 with NAFLD [10,137]. In a double-blind RCT (NCT01650025), in which were enrolled 48 pediatric NAFLD, 4 months VSL#3 supplementation improves severity of NAFLD as a consequence of VSL#3-induced eubiosis [138]. Administration of *Bifidobacterium longum* along with the prebiotic fructo-oligosaccharides (FOS) strongly improved circulating metabolic and inflammatory markers and fibrosis scores in patients with biopsy-proven NASH [139]. Intriguingly, the presence of *Bifidobacterium longum* in the VSL#3 compound modulates gut microbiota to produce conjugated linoleic acid (CLA), which, in turn, affect fatty acid composition in the liver further corroborating that the interplay between the gut and the liver in NAFLD assumes a relevant role for the development of therapeutic interventions.

In a phase I study, consumption of *Lactobacillus* GG for 8 weeks induced changes in gut microbiome of cirrhotic patients with minimal hepatic encephalopathy (*n* = 30), which reduced the abundance of Enterobacteriaceae, endotoxemia and TNF-α and facilitated the intestinal growth of Clostridiales Incertae Sedis XIV and Lachnospiraceae [140]. Recently, Romàn et al. evaluated the effect of a mixture of eight strains for 12 weeks in 36 patients with cirrhosis and found that the multistrain probiotic improved cognitive function, lower the incidence of falls at the follow-up and decreased inflammatory responses [141]. Conversely, in a randomized double-blind RCT including 44 cirrhotic outpatients, the long-term probiotic supplementation consisting of *Bifidobacterium bifidum* W23, *Bifidobacterium lactis* W52, *Lactobacillus acidophilus* W37, *Lactobacillus brevis* W63, *Lactobacillus casei* W56, *Lactobacillus salivarius* W24, *Lactococcus lactis W19 and Lactococcus lactis* W58, significantly influenced neutrophil resting burst, but not circulating endotoxins, gut permeability or inflammatory markers [142]. Although many of the current studies on effectiveness of probiotics are disappointing, this preliminary data has suggested that they are a well-tolerated and safe in cirrhotic subjects.

Few studies regarding the use of probiotics as HCC therapy have been reported in humans. Remarkably, it has been observed that preoperatively and postoperatively probiotic supplementation in patients with HCC who underwent hepatic resection favored liver function recovery and showed reduced frequency of intraoperative and postoperative complications [143].

## 5. Focus on Clinical Trials Regarding NAFLD and Probiotics

The effect of probiotics on NAFLD development and on metabolic syndrome is currently investigated in larger and long-term clinical trials. Indeed, the impact of the dietary supplementation for 6 months with *Lactobacillus acidophilus* ATCC SD5221 and *Bifidobacterium lactis* HN019 on hepatic changes in NASH patients is under definition in the single-center double blind, placebo controlled, parallel group study NCT02764047 [134,137,144,145]. The main outcomes of this study are the noninvasive evaluation of fibrosis, AST, ALT, the restoration of gut microbiota and the amelioration of the lipid profile via the measurement of circulating total cholesterol, HDL, LDL and triglycerides. The effectiveness of the probiotics in improving the liver functions is also the topic of the randomized, double-blind, placebo-controlled trial NCT04074889 of 6 months duration. This study assessed hepatic inflammation, fibrosis and intestinal permeability in NAFLD patients after the administration of a mixture of *Lactobacillus* and *Bifidobacterium* containing probiotics (*Lactobacillus acidophilus*, *Lactobacillus casei* subsp, *Lactobacillus lactis*, *Bifidobacterium bifidum*, *Bifidobacterium infantis* and *Bifidobacterium longum*). The randomized interventional study NCT01922830 tested the dietary supplementation to NAFLD obese subjects who underwent bariatric surgery with a mixture of different bacterial species (in the formulation of Bio-25 (Supherb) consisting of 11 different species of patented probiotic bacteria). Likewise, even in NCT03585413 it has been evaluated the effect of gut microbiota manipulation and the mini-gastric bypass surgery in the contest of obesity. Moreover, phase II of NCT03511365 clinical trial investigated the alteration of serum inflammatory markers and fecal microbiota following the administration of VSL#3 in patients with NAFLD. Finally, NCT03511365 and NCT02972567 are currently recruiting patients with NASH with the aim to ameliorate hepatic inflammation and fibrosis and to improve the prognosis of these patients and their cardiovascular risk, after intestinal microbiota restoration.

The efficacy of probiotics in metabolic disease remains a matter of debate and probably it necessitates further investigations to evaluate their safety, doses and short/ long-term exposure and the benefits to be administered alone or in combination with current therapy for NAFLD.

A schematic description of the main ongoing clinical trials is represented in Table 1.

## 6. Conclusions

Through different routes, gut microbiota composition and function are strongly entangled in the pathogenesis and the progression of liver injury in patients with metabolic syndrome and NAFLD, the major health concerns in children and adult population. In particular, intestinal bacterial overgrowth, dysbiosis and intestinal barrier derangement along with several other issues concur to increase individual susceptibility to NAFLD. Further studies are essential to completely draft the true causality between changes observed in the context of NAFLD and comorbidities as well as to pinpoint the mechanisms through which microbiota alterations affect liver pathology. To date, clinical guidelines indicate dietary interventions and lifestyle modifications as a gold standard for the treatment of NAFLD and its comorbidities, although the discouraging results due to the poor compliance of patients. Therefore, there is an urgent need to identify alternative therapeutic strategies to tailor NAFLD management.

Several studies and clinical trials have encouraged the use of probiotic supplementation as promising and safe therapeutic approach, highlighting the uncharted avenue of the intestinal microflora restoration as a cornerstone in the standard of clinical care of NAFLD patients. Trustworthily, addressing gut microbiota as a new driving direction for the future medicine will brighten the avenue of personalized interventions. Nevertheless, microbiota composition investigation could become an appealing candidate even for diagnosis, attempting to profile liver disease stage. In addition, probiotics could be administered alone or in a combination with NAFLD current therapies although their synergic effects remain largely unexplored.

To date, the efficacy of probiotics in NAFLD management is unknown and limited to hypotheses. However, extensive studies are essential to completely pinpoint the mechanisms through which microbiota alterations affect liver pathology in NAFLD patients and to identify the most effective probiotic strains, their doses, timings and the duration of administration.

## Figures and Tables

**Table 1:** Clinical trials underway addressing the therapeutic modulation of gut microbiota in NAFLD patients.

Clinical Trial Start-End Date	Status	Study Type	Interventions	Conditions	Objectives	Locations
NCT02764047 04/15–12/17 [134,137,144,145]	Recruiting (*n* = 58) *	Interventional Randomized	10^9^ *Lactobacillus acidophilus* ATCC SD5221 and 10^9^ *Bifidobacterium lactis* HN019 vs. placebo	NASH	Evaluate the effect of supplementation of probiotics on liver changes (histological and enzymatic), lipid profile and gut microbiota	Federal University of Health Science of Porto Alegre
NCT03528707 04/15–05/18	Completed (*n* = 48)	Interventional Randomized	Dietary Supplement Symbiter Omega for 8 weeks vs. placebo	T2DM with NAFLD	Assess the impact of co-administered multi-strains probiotic and omega-3 on steatosis, lipid profile and inflammation	Bogomolets National Medical University
NCT01922830 08/13–01/19	Active, not recruiting (*n* = 100) *	Interventional Randomized	Dietary Supplement: Bio-25 (Supherb) vs. mimic Bio-25 pill	NAFLD patients undergoing sleeve gastrectomy surgery	Investigate the benefits of 6 months probiotic supplement on clinical and metabolic parameters in patients with NAFLD Bariatric Surgery	Tel-Aviv Sourasky Medical Center
NCT04074889 08/19–12/20	Recruiting (*n* = 48) *	Interventional Randomized	Microbial cell preparation (Hexbio) for 6 months vs. placebo sachet with no microbial cell preparation	NAFLD	Evaluate intestinal barrier function, local gut inflammation and the clinical outcomes in NAFLD patients.	Universiti Kebangsaan Malaysia Medical Centre
NCT03511365 05/18–08/19	Enrolling by invitation (*n* = 20) *	Interventional Single Group Assignment (phase II)	VSL#3 vs. placebo	NAFLD	Examine the alterations in serum inflammatory markers and fecal microbiota after VSL#3 supplementation	Northwell Health, Manhasset, New York, United States
NCT03467282 03/18–08/19	Recruiting (*n* = 46) *	Interventional Randomized	1g probiotic mix (twice day) vs. 1g polydextrose/maltodextrin	NASH	Analyze the microbiota modulation, degree of hepatic steatosis, inflammation and fibrosis, and body composition	Hospital de Clinicas de Porto Alegre Porto Alegre, RS, Brazil
NCT03585413 08/18–08/21	Recruiting (*n* = 60) *	Interventional Randomized (phase III)	micronutrient-probiotic-supplement vs. placebo	Obese patients undergoing to mini-gastric bypass surgery	Investigate the effect of probiotic on fatty liver, IR, NAFLD/NASH progression and cardiometabolic diseases.	St. Franziskus-Hospital Cologne, Germany
NCT02972567 10/16–06/17	Recruiting (*n* = 60) *			Metabolic Syndrome X	Assess changes in intestinal microbiota, lipid profile, markers of inflammation, hypertension, cardiovascular risk and hepatic steatosis.	Complejo Hospitalario Universitario de Jaen, Jaen, Spain
Interventional Randomized (phase II)	1 capsule/day of *Lactobacillus spp* for 12 weeks vs. maltodextrin					

* Estimated number of participants.

## Abbreviations

α-SMA	α-smooth muscle actin
ADH	Alcohol Dehydrogenase
ANGPTL4	Angiopoietin-like 4
ALD	Alcoholic Liver Disease
ALT	Alanine Aminotransferase
AST	Aspartate Aminotransferase
BMI	Body Mass Index
CCL	Chemokine C-C motif Ligand
CCL4	Carbontetrachloride
CDAA	choline-deficient/L-amino acid-defined diet
CDCA	chenodeoxycholic acid
CLA	Conjugated Linoleic Acid
COX-2	Cyclooxygenase 2
CYP2E1	Cytochrome P450 2E1
Cyp7A1	Cholesterol 7-α hydroxylase 1
DAMPs	Damage-Associated Molecular Patterns
DCs	Dendritic cells
DEN	Diethylnitrosamine
DCA	Deoxycholic Acid
ER	Endothelial Reticulum
FFAs	Free Fatty Acids
FGF19	Fibroblast Growth Factor 19
FIAF	Fating-Induced Adipocyte Factor
FMT	Fecal Microbiota Transplantation
FOS	Fructo-oligosaccharides
FXR	Farnesoid X Receptor
GIT	Gastrointestinal Tract
HCC	Hepatocellular Carcinoma
HDL	High Density Lipoprotein
HFD	High Fat Diet
HFGFD	High Glucose/Fructose Diet
HOMA-IR	Insulin Resistance Index
HSHF	High Sucrose and High Fat
HSCs	Hepatic Stellate Cells
IFN-γ	Interferon-γ
IgA	Immunoglobulin A
IL	Interleukin
iNOS	Inducible nitric oxide synthase
IR	Insulin Resistance
JNK	Jun N-terminal kinase
LDL	Low Density Lipoprotein
LP-F19	Lactobacillus Paracasei F19
*Lep* ^ob/ob^	Leptin Deficient Mice
LPL	Lipoprotein Lipase
LPS	Lipopolysaccharides
MBOAT7	Membrane Bound O-acyltransferase Domain-containing 7
MCD	Methionine-Choline Deficient Diet
MetS	Metabolic Syndrome
miRNAs	microRNAs
MMP	Metalloproteinases
MTT	Microbiota-Targeted Therapy
Myd88	Myeloid differentiation factor 88
NAFLD	Nonalcoholic fatty liver disease
NASH	Nonalcoholic steatohepatitis
NF-κB	Nuclear Factor Kappa-Light-Chain-Enhancer Of Activated B Cells
NLR	NOD-like receptors
NO	Nitric Oxide
NOD	Nucleotide-binding and Oligomerization Domain
PAMPs	Pathogen-Associated Molecular Patterns
PNPLA3	Patatin-like Phospholipase Domain-containing 3
PPAR- γ	Peroxisome Proliferator-Activated Receptor-γ
qRT-PCR	Quantitative Real Time Polymerase Chain Reaction
RCT	Randomized Placebo-Controlled Trial
Reg3b	Regenerating islet-derived protein 3 b
Reg3g	Regenerating islet-derived protein 3 g
ROS	Reactive Oxygen Species
rRNA	Ribosomal RNA
SASP	Senescence-Associated Secretory Phenotype
SCFA	Short Chain Fatty Acid
SNPs	Single nucleotide polymorphisms
T2DM	Type 2 Diabetes Mellitus
TGF- β	Transforming growth factor β
TGR5	Takeda G-protein-coupled receptor 5
Th17	T-helper cells 17
TLR	Toll-like receptor
TMAO	Trimethylamine N-oxide
TM6SF2	Transmembrane 6 Superfamily Member 2
TNF-α	Tumor Necrosis Factor alpha
VLDL	Very-Low Density Lipoprotein
UCP-2	UCP-2
WHO/FAO	World Health Organization/Food and Agriculture Organization
ZO-1	Zonula Occludens-1
